# The Raven’s Coloured Progressive Matrices in Healthy Children: A Qualitative Approach

**DOI:** 10.3390/brainsci10110877

**Published:** 2020-11-20

**Authors:** Daniela Smirni

**Affiliations:** Department of Psychology, Educational Science and Human Movement, University of Palermo, 90128 Palermo, Italy; daniela.smirni@unipa.it

**Keywords:** intelligence, RCPM qualitative parameters, attentional abilities, analogical reasoning, concrete and abstract reasoning

## Abstract

Studies on the structure of intelligence refer to two main theoretical models: the first one considers intelligence as a unitary construct, the second one assumes the involvement of a plurality of factors. Studies using Raven’s Coloured Progressive Matrices (RCPM) tasks have often highlighted the involvement of different cognitive abilities and brain structures, but in the clinical setting, RCPM measurement continues to be used as a single score. The current study aimed to analyse the RCPM performance following qualitative clustering, in order to provide an interpretation of the intelligence assessment through a factorial criterion. The RCPM have been administered to a large group of typically developing children between 6 and 11 years of age. The sample was divided into twelve age groups, and the performance of each age group was shown. Three qualitative item clusters were identified through a factorial criterion. Data showed that performance on RCPM may arise from different cognitive abilities, starting from basic attentional skills up to the more complex ones that require perceptual or analogical reasoning. The qualitative parameters could provide more effective diagnostic and treatment suggestions than a single total score in the clinical setting, and may be crucial for focusing on strength and weakness in the intellectual functionality evaluation of children.

## 1. Introduction

Most theories on the nature and the structure of intelligence can be summarised by two broad theoretical models: the first one considers intellectual activities as the expression of a unitary construct, and the second hypothesises a plurality of factors with different primary mental abilities that are functionally independent [1]. 

In the early decades of the last century, on the basis of statistical–mathematical methods, Spearman proved that intelligence tests scores, although different, correlate positively with each other. However, he noted that the strength of these correlations often was not strong. Therefore, he decomposed the variance in test scores to the part that is common with all other tests, as well as to the part that is unique to the given test [2]. With this background, Spearman hypothesised that each test measures a general ability, which he labeled as the “*g* factor”, and a specific factor tapping abilities or processes unique to the given test, which he labeled as the “*s* factor” [3,4]. In this context, intelligence, as a “*g* factor”, could be mediated by a specialised region of the brain or by the whole brain being globally activated [5].

Subsequently, the further development of factorial analysis methods has encouraged other structural theories of intelligence, mainly on a statistical basis. Burt [6], for example, hypothesised a hierarchy of intellectual abilities, and distinguished in each individual test three orders of factors: general, group, and specific factors. Thurstone [7], using factorial analysis, concluded that there is no general factor, but many lower-order, uncorrelated modules or neural processes (so-called bonds), while Thomson [8] rejected Spearman’s *g* because the positive manifold should emerge without a single general ability, through co-sampling. Guilford [9] expanded Thurstone’s multifactorial theory, using parameters to classify the high number of isolated intellectual abilities (about 120). On this basis, it could be hypothesised that different brain regions mediate distinct and relatively independent intellectual functions [5].

From then to now, new theoretical accounts have been proposed to explain the positive correlation between tests, and the debate remains an outstanding issue [10], although its articulation exceeds the limits of this study. 

Some models proposed a theoretical framework in which the singularity and plurality of factors seem to be connected. For example, the Cattel–Horn–Carrol theory combines the multifactor theory, including fluid and crystallised intelligence, and a hierarchical theory [11,12,13,14].

Recently, a “mutualistic” dynamic model has been developed to explain the positive manifold pattern. This model assumes that the cognitive system consists of many basic processes, such as perception, memory, decision, and reasoning. These processes, initially unrelated, interact with each other during development, becoming a correlated mutualistic network structure in which each process favours the development of the others. Therefore, there is no single underlying *g* factor, but rather a positive interaction between cognitive processes during development that gives rise to the positive manifold [15,16].

The recent process–overlap theory [17] highlights that intelligence is determined by multiple components, both domain-general and domain-specific, and that certain domain-general processes overlap with domain-specific processes during mental test performance. The domain-general processes are tapped by a large number of tests, while specific processes are mostly tapped by tests with corresponding specific (verbal, spatial, etc.) content only. For example, domain-general attention modulation allows for stimulus focus and interference inhibition. This mechanism seems to be a common component of all valid tests of working memory capacity (regardless of the domain) [18]. In this context, Intelligence Quotient (IQ) seems to be redefined as an index of specific cognitive abilities, rather than as an expression of a latent general cognitive ability [17].

Even today, then, there is a contentious debate about the right level of test score interpretation, both in clinical assessments and in research settings. In tests like the Wechsler scales, for example, some authors argue for a profile-based interpretation of subscale or even subtest scores, especially when the verbal and performance IQ or Verbal Comprehension Index and Perceptual Organization Index scores are extremely or significantly different [19,20,21,22].

Clinicians take the single full-scale IQ (FSIQ) less into account in favour of a complex cognitive profile that highlights specific abilities to look for the patient’s cognitive strengths and weaknesses [23,24]. Fiorello and colleagues, in a direct comparison of typical and atypical populations, found that FSIQ does not adequately represent global intellectual functioning for either typical children with significant profile variability or children with disabilities, suggesting that FSIQ should be de-emphasied as a measure of ability and greater emphasis should be placed on index scores, especially when significant index score differences are evident [23].

Conversely, many researchers argue against the validity and utility of profile interpretation and claim that global scores, such as FSIQ, are the most appropriate for diagnostic interpretation [25,26,27,28,29]. FSIQ would be the simplest and most efficient method of Wechsler Intelligence Scale for Children (WISC) analysis, and a more useful measure than index scores in the prediction of academic achievement [26,28,29,30].

Taken together, therefore, the studies presented compelling evidence against both the uniform interpretation of global FSIQ and cognitive profiles, suggesting that the conception of intelligence test results should be linked to an intelligence model. 

The Raven Progressive Matrices test, both in its usual form [31] and in the simplest coloured form, the Raven’s Coloured Progressive Matrices (RCPM) test [32], are widely considered as the most specifically designed test to measure the *g* factor and as the purest measure of fluid intelligence [33]. They were developed in the 1930s as a “culture-free” and nonverbal test to study the genetic and environmental determinants of “general” intelligence, defined as the ability to identify logical relationships within different elements and to generate the abstractive rules to organise them [34]. Spearman, in fact, differentiated two sources of the *g* factor: “eductive” and “reproductive” ability. The Raven’s Matrices were designed to measure the former, and their companion test, the Mill Hill Vocabulary Scales, were designed to measure the latter [35].

Over the years, a large number of researchers have also used RCPM with focal brain damage, leading to conflicting results. Some authors found poorer performances in left-brain damaged patients [36], others in the right-brain damaged patients [37,38]. Basso et al. [39] focused on the main roles of the two brain areas that would support performance on such a test: an area in the anterior left hemisphere, partially overlapped with the language areas, and a region in the posterior right hemisphere. 

To explain such disparity between the results, Costa [40] pointed out that RCPM would not have a homogeneous laterality index across the three sets (Sets A, Ab, B). Each set would include items requiring different cognitive abilities. The first set (Set A) would require predominantly visuoperceptual abilities; the second and third sets would involve mainly configuration processing (Set Ab) and analogical reasoning (Set B). 

Denes et al. [41] showed selectively impaired performances on each set after left or right brain damages. Zaidel and colleagues [42], in commissurotomy and hemispherectomy patients, found that the “general intelligence”, as measured by RCPM, is bilaterally represented in the cerebral cortex, but in unequal amounts for the different parts of the test, and hypothesised that two different *g* factors (left and right) may need to be distinguished. Some years later, three different clusters of items, based on the specific criterion required to provide the correct answer, were used [43]. Moreover, patients with a right hemispheric lesion have shown more difficulties in visuospatial tasks and, conversely, patients with left lesions have had worse performance in tasks requiring verbal reasoning [44]. 

Since 1974, Hunt [45] provided a theoretical account to problem-solving in Raven’s Advanced Progressive Matrices, proposing two qualitatively different solution algorithms: (1) a gestalt–perceptual algorithm, based on visual perceptual operations; and (2) an analytical picture based on feature representations and logical operations. Later, several factorial analysis studies have identified different factors presented among Raven test items (gestalt, analytic, spatial, verbal, and so on), supporting the hypothesis that different cognitive abilities may be involved during the problem-solving process tested by Raven Matrices tasks [46,47,48,49,50,51]. 

Carpenter et al. [46], for example, identified five rules used to solve all items in the Advanced Raven Matrices, and proposed a computer simulation model that can find the relevant rules in an item. Similarly, Lovett and Forbuss [52], claiming that analogical reasoning is crucial in the visual problem solving, devised a computational model to solve visual problems from the Raven’s Progressive Matrices.

Two different factorial studies [53,54] on RCPM, using different coefficients, extracted three factors: Factor I is pattern completion through identity and closure, Factor II is closure and abstract reasoning by analogy, and Factor III is simple pattern completion. Raven himself, however, suggested that various sets of items require simple pattern completion, discrete pattern completion, pattern completion with closure, concrete reasoning by analogy, and abstract reasoning by analogy [55]. The same global score, therefore, may result from different cognitive abilities.

More recently, neuroimaging studies have reported that items involving analytic reasoning tend to activate bilateral frontal areas and left temporal, parietal, and occipital regions, while elements involving visuospatial reasoning activate right frontal and bilateral parietal brain areas [56,57,58,59,60,61,62]. Taken together, lesion, factorial analysis, and neuroimaging studies seem to highlight the involvement of different cognitive abilities and different brain structures in the RCPM tasks.

The present study aimed to verify, in a large sample of healthy children, whether the clustering of items, according to factorial structure devised in previous literature studies, could provide more effective diagnostic and treatment suggestions than a single total score. 

The hypothesis was that a clustering of items according to three factors, extracted in literature, could provide a more clinically significant profile of the different cognitive abilities involved in RCPM in each age group.

## 2. Materials and Methods

### 2.1. Participants 

As part of a neurodevelopmental assessment, a survey for healthy primary school students was performed. The survey was aimed at investigating the neurodevelopment of cognitive functions in healthy children, and it was approved by the local ethical committee and school board. It was conducted according to the ethical principles of the Helsinki Declaration. Only children whose parents had given informed consent were examined. Students or parents who did not agree to take the exam were excluded from the study. 

A sample of 947 healthy children participated in the study, 476 boys and 471 girls, subdivided in 12 age groups ranging from 6 to 11 years. The number of subjects in each group and the male/female distribution were counterbalanced across the groups. Participants were recruited from the population in 13 Sicilian municipalities; they were volunteers for participating in the survey; of medium social status; and they had no history of neurological or psychiatric diagnosis, learning disability, or developmental delay. 

### 2.2. Procedures

The RCPM was administered individually, without time limit, in the book format, according to Raven’s procedure [63]. Children were asked to choose the missing element from six options in a drawing. One point was given for each correct answer, and the total score was the sum of the correct answers, with a maximum score of 36. 

### 2.3. Statistical Analysis

A qualitative analysis of the items, based on the cognitive abilities required on each item, was performed.

Following the analysis of the existing literature, the factorial structure of the RCPM was presented on the basis of criteria that grouped the items through the cognitive processes involved.

The sample was divided into twelve age groups, spaced 6 months from each other, and the performance on each age group was calculated according to the factor clustering, on which mean scores and standard deviations of correct answers were calculated. The independent *t*-test was used to compare mean scores between age groups.

In addition, mean and standard deviation values were also calculated according to a breakdown of the sample by age and sex.

## 3. Results 

A qualitative analysis of the cognitive abilities involved in each item of the RCPM is summarised in Table 1. Some items require only the completion of a pattern on the basis of similarity and identity (light grey background in Table 1). Other items require additional skills that are more complex than simple identity, such as the pattern completion based on the closure principle of a configuration, the directionality of the elements (medium grey background in Table 1), or high abstract reasoning (dark grey background in Table 1).

Table 2 shows the factorial structure of the RCPM extracted by factorial studies in the literature [53,54]. Factor I, called “continuous and discrete pattern completion through closure”, was described by items A7, A8, A9, A10, Ab4, Ab5, Ab6, Ab7, Ab8, Ab9, Ab10, Ab11, B3, B4, and B5. Factor II, called “closure and abstract reasoning”, was identified by items A11, A12, Ab12, B6, B7, B8, B9, B10, B11, and B12. Factor III, called “simple pattern completion”, consisted of items A1, A2, A3, A4, A5, A6, Ab1, Ab2, Ab3, B1, and B2. 

The mean scores and standard deviations of the correct answers of the 12 age groups, considering the item clustering by the three factors, are displayed in Table 3. 

Analysing the results, according to the three factorial clusters, the factor III items were almost completely solved from the age of six (six-years-olds’ mean score = 10.76 ± 0.72 vs. 11.5-year-olds’ mean score = 10.91 ± 0.19, out of 11 items) with no significant difference between the mean scores of the younger and older groups (*t*_148_ = 1.77, *p* = 0.08). Similarly, very low standard deviations were detected in all the groups. Such data proves that the cognitive abilities implied in factor III already at the age of six in normal children are sufficiently developed, and that from this age those abilities do not discriminate between groups.

Instead, in both factors I and II, the results were largely lower than for factor III, but the performance gradually increased with increasing age. Factor I six-year-olds’ mean score was 8.15 ± 2.83, versus 11.5-year-olds’ mean score of 14.13 ± 1.20, of the 15 items (*t*_148_ = 17.002, *p* < 0.001). Factor II six-year-olds’ mean score was 1.88 ± 1.71, versus 11.5-year-olds’ mean score of 5.69 ± 2.15, out of 10 items (*t*_148_ = 11.63, *p* < 0.001). However, while in factor I, the oldest group reached a score very close to the maximum, in factor II, no group reached the maximum score, and the lowest scores were recorded.

Considering the performance of the individual age groups, all groups reached the best scores for factor III, with scores very close to the maximum values, while they reached lower scores for factor I and even lower scores for factor II. Therefore, the clustering of items according to factor I, and even more the clustering according to factor II, proves to be more difficult, while factor II was the most difficult for each age group, suggesting that the cognitive abilities implied in these groups of items become discriminatory in the assessment of intellectual abilities examined in the RCPM.

Finally, no difference was found between the mean scores of males and females in any age group (Table 4).

## 4. Discussion 

The present study aimed to verify whether a clustering of items, based on previous factorial studies, could allow, in clinical setting, more effective diagnostic and treatment suggestions than the overall score.

Generally, in daily clinical practice, RCPM test is widely used as one of the best general intelligence measures, and it provides a single total score. In such a scoring system, one point is assigned to any correct item, regardless of the cognitive process involved in that specific single item. It seems, therefore, that the right answers exclusively result from a single factor, like Spearman’s general intelligence. Conversely, a single total score does not consider that each item may have different cognitive features and may be supported by different cognitive abilities. That is, the same result can derive from responses resulting from different qualitative clusters, and therefore underlie different cognitive abilities. This analysis of the type of cluster involved may have different diagnostic and clinical relevance.

In the present study, a large group of typically developing children were administered RCPM tests according to the standard procedure. Three qualitative clusters of items were identified according to factorial studies of literature. The results of the sample were examined using the three qualitative clusters, factorially extracted. 

As expected, in each cluster, scores gradually increased with increasing age. However, it is interesting to point out that in the factor III cluster, implying simple or discrete pattern completion, the problems have been solved properly by almost the whole sample in every age groups. Therefore, cluster items proved relatively simple as early as the age of six, probably because they are supported by basic cognitive skills. These findings may be particularly suggestive in the clinical evaluation of general cognitive function. Poor performance in such items may have a relevant clinical and treatment prominence, suggesting an impairment in basic cognitive abilities. Similarly, any poor total score resulting from poor performance in the problems of the factor III cluster should have a different diagnostic value than a poor total score resulting from other combinations of wrong items.

Factor III includes items in the form of a continuous pattern, where the missing piece and pattern have the same formal visuo-spatial features (e.g., A4, A5, Ab1, Ab2, Ab3, B1, B2). That is, the choice of the entry is mainly mediated by selective attentional analysis, as well as visual discrimination and visual matching processes. Therefore, the wrong answers might reflect poor selective attention and immediate focusing on a single, main, prominent perceptual element. The factor III items evaluate the basic skills of attentional and visuo-perceptive exploration, in terms of identity, similarity, and orientation correspondence. Six-year-old children proved able to identify the missing element to complete a continuous or discrete pattern, even in the presence of alternative choices as distractors, and from an early age, visuo-spatial attentive abilities implied in attentive problems of this cluster items appears sufficiently organised. These findings are consistent with a previous study [64] that showed that children from the age of nine and up performed almost as well as the adult control group in a complex visual discrimination tasks, such as Benton’s Visual Form Discrimination Test [65]. 

In the factor I cluster, implying continuous and discrete pattern completion and pattern completion through closure, the results in all age groups were poorer than for factor III, and no age group reached the maximum scores, although by increasing the age the results gradually improved. As a whole, then, this item cluster proved to be more complex and demanding. The choice of missing element in this item cluster implied an increasingly more complex perceptual gestalt process, requiring a selective analysis of the single elements and a shifting from part–whole and whole–part to form a coherent gestalt and consistent global whole (e.g., A7, A8, Ab4, Ab5, B3, B4). This cluster of items evaluates integrated perceptual abilities involved in the closure of a coherent whole or gestalt through the analysis of the “whole” and “parts”.

In the items of factor II, involving closure as well as concrete and abstract reasoning by analogy, the performances were the poorest in all the age groups, especially in the younger ones. Group performance improves with increasing age, but no group has achieved full scores. Even the older groups showed a still-incomplete maturation of the abstractive functionality of the prefrontal regions involved in these kinds of tasks [66,67]. This cluster includes homogeneous items in which the detection of the missing piece implies a higher abstract analogical process. To understand similarities and differences between the elements, a target figure and alternative choices must be simultaneously analysed in their abstractive–analogical relationship [68]. Such processes involve the functional integrity of cortical and subcortical networks—like the prefrontal and posterior parietal regions [68], that underlie reasoning and simultaneous processing of more stimuli [44]—and show the slowest development, being still inadequate and immature before the age of 12 [69,70].

The wrong choices, then, result from concrete thinking using only perceptual or similarity criteria, due probably to reduced prefrontal functionality and connectivity [71]. 

## 5. Conclusions

The current study showed that RCPM items are not homogeneous tasks. The same scoring may be supported by different cognitive abilities and different clusters of correct items, and it may have a different diagnostic and clinical relevance.

Therefore, RCPM should be reorganised, taking into account the different cognitive skills involved in the various item clusters, and should be analysed according to qualitative criteria, as attentional, perceptual, and analogical clusters.

Two strong clinical implications may derive from this study. In the neuropsychological assessment of children, the three qualitative clusters can provide more functional clinical parameters on basic attentive abilities, as well as perceptual and analogical reasoning, which can be crucial to focusing on strengths and weaknesses for the targeted vocational and treatment programs. 

In addition, since performance on the RCPM arises from multiple working memory systems [72], a qualitative analysis may provide significant vocational suggestions and predict how well many different activities will be performed.

Finally, this study could also support a short form of the RCPM [73]. Removal of items that already show a ceiling effect around the age of six, and which do not discriminate by age, could favor a faster but equally effective neuropsychological assessment, as documented by short forms of memory tests [74,75].

A limitation should be considered. The current study focused only on a large sample of typically developing children, but it provides useful data on healthy subjects expressed in terms of mean scores and standard deviation values, which can be used to compare the performance of clinical subjects of the same age on the basis of this aggregation of items. However, further research is needed to study the qualitative approach on intelligence evaluation in several clinical neurodevelopmental populations. 

## Figures and Tables

**Table 1:** Qualitative analysis of items: cognitive processes involved in each Raven’s Coloured Progressive Matrices (RCPM) item.

Item	Cognitive Processes Involved
A2	Difference, identity
A3	Difference, identity, similarity
B1	Difference, identity, similarity
A1	Difference, identity, similarity
A4	Difference, identity, similarity
A5	Difference, identity, similarity
Ab1	Difference, identity, similarity
B2	Difference, identity, similarity
Ab3	Identity, similarity, identical orientation
Ab2	Identity, similarity, identical orientation
A6	Difference, similarity, identical orientation
B3	Similarity, symmetrical orientation, closure
B4	Similarity, symmetrical orientation, closure
Ab4	Similarity, symmetrical orientation, closure
Ab5	Similarity, symmetrical orientation, closure
Ab7	Similarity, symmetrical orientation, open symmetry
A9	Difference, identity, similarity, symmetrical orientation
A10	Difference, identity, similarity, symmetrical orientation
A7	Difference, similarity, gestalt of completion
Ab6	Closed asymmetry, symmetrical orientation, gestalt closure
B5	Similarity, asymmetry, orientation towards closure
A8	Difference, similarity, gestalt of completion
Ab9	Open symmetry, symmetrical orientation
Ab8	Closed asymmetry, orientation towards closure
Ab10	Open symmetry, symmetrical orientation
Ab11	Closed symmetry, symmetrical diagonal orientation
B6	Asymmetrical change, asymmetrical diagonal orientation
B7	Asymmetrical change, asymmetrical diagonal orientation
B10	Elements addition to a changing figure
A11	Difference, similarity, gestalt of completion
B9	Asymmetrical change in a modified figure
B11	Elements subtraction to a changing figure
Ab12	Open asymmetry, asymmetrical orientation, response creation through analogy
B8	Asymmetrical change in a modified figure
A12	Similarity, orientation, response creation through analogy
B12	Double subtraction of specific characteristics to a figure

Items are displayed in descending order of the mean scores. Light grey background: completion on the basis of similarity and identity. Medium grey background: completion based on closure and directionality. Dark grey background: high abstract reasoning.

**Table 2:** Carlson and Jensen [53] and Wiedl and Carlson [54] factorial structure of RCPM.

Item	Cognitive Abilities	Factor
A1	Simple pattern completion	III
A2	Simple pattern completion	III
A3	Simple pattern completion	III
A4	Simple pattern completion	III
A5	Simple pattern completion	III
A6	Simple continuous pattern completion	III
A7	Pattern completion showing progressive change	I
A8	Pattern completion showing progressive change	I
A9	Pattern completion showing progressive change	I
A10	Pattern completion showing progressive change	I
A11	Concrete and abstract reasoning	II
A12	Concrete and abstract reasoning	II
Ab1	Pattern completion involving identity	III
Ab2	Pattern completion involving identity	III
Ab3	Pattern completion involving identity	III
Ab4	Pattern completion through closure	I
Ab5	Pattern completion through closure	I
Ab6	Pattern completion through closure	I
Ab7	Pattern completion through closure	I
Ab8	Pattern completion through closure	I
Ab9	Discrete pattern completion	I
Ab10	Discrete pattern completion	I
Ab11	Discrete pattern completion through closure	I
Ab12	Abstract reasoning by analogy	II
B1	Pattern completion involving identity	III
B2	Pattern completion involving identity	III
B3	Pattern completion involving identity and directionality	I
B4	Pattern completion through closure	I
B5	Pattern completion through closure	I
B6	Concrete and abstract reasoning	II
B7	Concrete and abstract reasoning	II
B8	Concrete reasoning by analogy	II
B9	Concrete reasoning by analogy	II
B10	Abstract reasoning by analogy	II
B11	Abstract reasoning by analogy	II
B12	Abstract reasoning by analogy	II

**Table 3:** Mean and standard deviation of RCPM scores, according to the three-factorial clustering in each age group.

	Factor III	Factor I	Factor II	Total Score
Age Group	Mean ± SD	Mean ± SD	Mean ± SD	Mean ± SD
6.0	10.76 ± 0.72	8.15 ± 2.83	1.88 ± 1.71	20.75 ± 3.97
6.5	10.78 ± 0.64	8.38 ± 2.42	1.53 ± 1.35	21.05 ± 3.52
7.0	10.79 ± 0.61	8.85 ± 2.90	2.04 ± 1.88	21.99 ± 4.17
7.5	10.82 ± 0.67	9.48 ± 3.08	2.09 ± 2.01	22.66 ± 4.43
8.0	10.86 ± 0.55	10.75 ± 3.06	2.55 ± 2.19	24.35 ± 4.51
8.5	10.89 ± 0.46	11.36 ± 2.68	2.90 ± 2.05	25.46 ± 4.03
9.0	10.89 ± 0.33	12.00 ± 2.71	3.81 ± 2.47	26.81 ± 4.39
9.5	10.91 ± 0.34	12.65 ± 2.39	4.29 ± 2.56	27.84 ± 4.27
10.0	10.91 ± 0.25	13.17 ± 2.17	4.12 ± 2.36	28.26 ± 3.62
10.5	10.92 ± 0.19	13.15 ± 2.43	5.19 ± 2.67	29.32 ± 4.49
11.0	10.92 ± 0.33	13.47 ± 2.07	5.37 ± 2.65	29.78 ± 4.23
11.5	10.91 ± 0.19	14.13 ± 1.20	5.69 ± 2.15	30.81 ± 2.80
Overall	10.89 ± 0.44	11.36 ± 3.24	3.48 ± 2.62	25.85 ± 5.26

**Table 4:** Mean and standard deviation of RCPM scores in the three factors, according to a breakdown of the sample by age and sex.

			Factor III	Factor I	Factor II	Total Score
Age	Sex	*n*	Mean ± SD	Mean ± SD	Mean ± SD	Mean ± SD
6.0	F	35	10.78 ± 0.68	8.17 ± 3.05	1.91± 1.57	20.83 ± 3.84
	M	38	10.75 ± 0.85	8.13 ± 2.67	1.89 ± 1.92	20.74 ± 4.12
6.5	F	42	10.79 ± 0.59	8.38 ± 2.22	2.10 ± 1.80	21.19 ± 3.13
	M	36	10.75 ± 0.66	8.39 ± 2.54	2.08 ± 1.71	21.17 ± 3.10
7.0	F	38	10.82 ± 0.51	8.82 ± 2.82	2.13 ± 1.94	21.76 ± 3.84
	M	40	10.78 ± 0.69	8.85 ± 2.90	2.13 ± 2.00	21.75 ± 4.10
7.5	F	29	10.79 ± 0.74	9.41 ± 2.41	2.48 ± 2.18	22.62 ± 4.35
	M	36	10.85 ± 0.49	9.44 ± 2.93	2.50 ± 2.27	22.69 ± 4.65
8.0	F	37	10.86 ± 0.45	10.68 ± 3.27	2.81 ± 2.40	24.32 ± 5.04
	M	47	10.90 ± 0.49	10.72 ± 2.36	2.79 ± 2.32	24.38 ± 4.10
8.5	F	42	10.87 ± 0.53	11.29 ± 2.53	3.14 ± 2.13	25.33 ± 4.13
	M	39	10.91 ± 0.24	11.38 ± 2.41	3.13 ± 1.95	25.51 ± 3.69
9.0	F	33	10.95 ± 0.27	12.00 ± 2.74	3.85 ± 2.32	26.85 ± 4.43
	M	36	10.87 ± 0.45	12.00 ± 2.66	3.81 ± 2.41	26.78 ± 4.01
9.5	F	53	10.88 ± 0.45	12.62 ± 2.47	4.26 ± 2.59	27.87 ± 4.18
	M	47	10.92 ± 0.31	12.68 ± 1.83	4.19 ± 2.50	27.85 ± 3.65
10.0	F	54	10.89 ± 0.29	13.15 ± 2.51	4.13 ± 2.46	28.26 ± 4.07
	M	41	10.93 ± 0.22	13.20 ± 1.25	4.10 ± 2.03	28.27 ± 2.47
10.5	F	35	10.94 ± 0.15	13.11 ± 2.29	5.23 ± 2.72	29.34 ± 4.45
	M	33	10.90 ± 0.24	13.12 ± 2.38	5.24 ± 2.40	29.30 ± 4.25
11.0	F	38	10.95 ± 0.16	13.45 ± 1.92	5.39 ± 2.88	29.82 ± 4.17
	M	51	10.90 ± 0.41	13.49 ± 2.00	5.35 ± 2.52	29.75 ± 4.10
11.5	F	35	10.94 ± 0.17	14.14 ± 1.15	5.66 ± 2.14	30.80 ± 2.78
	M	32	10.89 ± 0.23	14.13 ± 1.17	5.72 ± 2.15	30.81 ± 2.84
Overall			10.89 ± 0.44	11.36 ± 3.24	3.48 ± 2.62	25.85 ± 5.26

Legend: F = Female; M = Male.

## Scoring

A scoring program based on the 3 factors is available on request at daniela.smirni@unipa.it.
